# Third-child fertility intentions and influencing factors among female workers of reproductive age in Shandong under China's three-child policy

**DOI:** 10.3389/fpubh.2025.1697298

**Published:** 2025-11-12

**Authors:** Yanxia Qi, Guiyun Wang, Jinke Kuang, Wenjing Xu, Yingxin Zhang, Xiuzhen Mu, Jinnan Xiao

**Affiliations:** 1 School of Nursing, Shandong Xiehe University, Jinan, Shandong, China; 2 Delivery Room, The Second People's Hospital of Liaocheng, Liaocheng, Shandong, China; 3 Taigong Primary School, Zibo, Shandong, China; 4 Xiangya School of Nursing, Central South University, Changsha, Hunan, China

**Keywords:** female workers, third-child fertility intentions, influencing factors, cross-sectional study, China's three-child policy

## Abstract

**Background:**

The persistent decline in global fertility rates, coupled with women's growing professionalization, has led to low third-child fertility intentions among female workers, a common challenge across low-fertility countries.

**Objectives:**

This study examined third-child fertility intentions and their influencing factors among reproductive-age female workers in Shandong Province, China.

**Methods:**

Using snowball sampling, 1,358 female workers aged 20–49 years in Shandong Province, China were recruited through personal networks from April–May 2024 and referrals to ensure a diverse yet targeted sample. Based on the theory of planned behavior, we collected data on demographic characteristics, behavioral attitudes, subjective norms, and perceived control factors using a self-administered questionnaire distributed through a popular online survey platform. We used descriptive analysis, chi-squared tests, and binary logistic regression for data analysis.

**Results:**

Only 10.3% of the study participants expressed intentions to have a third child. The logistic regression analysis showed that all examined variables—including demographic characteristics (age, residence, education level, monthly household income, and whether both spouses were only children), behavioral attitude factors (the ideal number of children and career prospects), and perceived control factors (caregiver availability for a third child, type of workplace, and knowledge of the three-child policy)—were significantly associated with third-child fertility intentions (*p* < 0.05). In terms of subjective norms, the factors exhibited no statistically significant associations (*p* > 0.05).

**Conclusions:**

Female workers face career–family conflicts and may avoid having a third child due to multiple factors. To achieve sustainable population development, China should intensify efforts to promote the three-child policy, clarify its benefits and implementation mechanisms, and address barriers to uptake, alongside expanding affordable quality public childcare, increasing childcare subsidies, and legislating family-friendly practices (e.g., flexible work) in private sectors. The study findings offer actionable insights for low-fertility regions with similar socio-cultural contexts, including other provinces in China and specific areas in East Asia.

## 1 Introduction

Childbearing is fundamental to the continuation of human beings and the establishment of kinship, ultimately providing the labor force needed for the development of human society. Fertility has declined steadily at the global level and across almost all countries and territories since 1950 and is likely to continue its declining trend until 2100 (1, 2). The continuous decline in fertility rates is a significant factor contributing to the aging global population (3). An increase in the older population and a decrease in the young population may place increasing burdens on health-care and social systems, transforming labor and consumer markets and hindering economic and social development (1, 4, 5).

China's large population accounts for nearly one-fifth of the global population, which has an important impact on global population trends. Therefore, China's population and fertility policies have attracted the attention of the world. In the 1970s, China implemented a family planning policy mandating one child per couple, which led to a declining fertility rate in China. The total fertility rate (TFR) decreased from 5.81 in 1970 to 2.75 in 1979 (6), and having only one child in a family has become the norm in Chinese society (7, 8). The fertility rate has declined to below the population replacement level since the 1990s, and the 2010 censuses across Chinese provinces yielded a TFR of 1.18 (9). In 2016, China officially implemented the Universal Two-Child Policy, allowing all couples to have two children, and the TFR rose to 1.3 in 2020. However, the fading effects of the two-child policy and the influence of coronavirus disease 2019 (COVID-19) led to China experiencing the lowest fertility rates in history, according to the results of the Seventh Census (3). To avoid the rapid aging of the population due to the continuous decline in the fertility rate, China started to implement a three-child policy in 2021 and further optimized it to promote long-term balanced development of the population, which included eliminating restrictive measures, such as social maintenance fees (fines for out-of-quota births), and removing related penalties to increase the willingness of couples of reproductive age to have a third child (10).

Despite the government's introduction of support measures for the three-child policy, fertility intentions among women of reproductive age in China remain relatively low. A study involving 61,588 women of childbearing age showed that only 5.01% of the sample had third-child fertility intentions (11). Other studies have also produced similar results, indicating that most Chinese women of childbearing age ideally want to have two children (12, 13). With the continuing development of society and women's increasing levels of education in China, the number and proportion of female workers are growing (14, 15). According to World Bank data, China's female labor force participation rate reached 62% in 2021, placing it among the top 20 economies globally, indicating that Chinese working women can greatly influence China's future social development. Studies have shown that working women have higher levels of social awareness and bear the dual burdens of family and work (14, 16, 17). Therefore, the desire to have three children among working women of childbearing age may differ from that of nonworking women. However, existing studies on China's three-child policy have rarely focused on reproductive-age female workers, resulting in incomplete understanding of how individual and societal factors shape their third-child intentions.

The Theory of Planned Behavior (TPB) was systematically elaborated by Ajzen (18) in his 1991 publication. The theory posits that behavioral intention is influenced by three core factors: behavioral attitudes, subjective norms, and perceived behavioral control. Since its proposal, the theory has been widely applied in the field of fertility research, enabling the prediction and explanation of fertility behaviors across different cultural contexts and policy environments. For instance, some studies have shown that fertility attitudes, subjective norms from family and peers, and the perceived control over economic and childcare resources can all effectively predict fertility decisions (4, 13, 19). Additionally, results from other studies have revealed that the TPB has been used to analyze fertility intentions under different fertility policies, revealing how cultural norms and policy constraints shape individual perceptions (11, 12, 20). Given that the TPB can effectively capture the underlying factors influencing fertility behaviors, we adopted this framework as the theoretical basis for our study.

Based on the above, we conducted a study in Shandong Province, in Central China, which is the birthplace of Confucian culture to explore third-child fertility intentions and their influencing factors among reproductive-age female workers. Findings aim to inform targeted fertility policies for this group and offer references for other low-fertility nations or regions.

## 2 Methods

### 2.1 Study design

We distributed a cross-sectional survey via electronic questionnaires to married female workers of reproductive age in Shandong Province to examine their third-child fertility intentions and to explore influencing factors.

### 2.2 Sample and setting

The survey was conducted from April to May 2024 in Shandong Province, which is located in eastern coastal China and has 16 prefecture-level administrative divisions. According to the 2020 National Census, Shandong had an urban permanent resident population of 101.5 million, ranking as the second-most populous province in China. The female population in Shandong was 50.1 million, with women aged 20–49 years accounting for 38.41% of the total female population, closely aligning with the national average for this demographic (41.93%) (21, 22).

For this study, we targeted female workers of reproductive age residing in Shandong who had 0–2 children at the time of data collection. Inclusion criteria were as follows: (1) married women, (2) age range 20–49 years old, (3) in paid work, (4) 0–2 current children, and (5) no serious physical or mental illness. We excluded participants who were pregnant with a third child, had undergone permanent sterilization procedures (such as tubal ligation or hysterectomy), had menopausal status according to the Chinese Guidelines for Menopause Management and Menopausal Hormone Therapy (2023 Edition; amenorrhea for ≥ 12 months), or had comorbid mental illnesses, cognitive impairments, or visual/auditory disorders that rendered them unable to provide valid information.

### 2.3 Sample size determination

Based on previous studies, the third-child fertility intention rate among women of reproductive age in China ranges from 5.01 to 14.4% (11, 23). Considering the influence of Confucian culture and concepts such as “more children, more blessings” in Shandong Province, we therefore assumed that the prevalence of third-child fertility intention was 10%. The sample size for this study was determined using Fisher's (1999) formula for estimating a population proportion: $n=\frac{z_{\alpha }^{2}\;*\;p\;*\;\left(1-p\right)}{\delta^{2}}$. In this formula, Z is the Z-statistic corresponding to the desired confidence level (set at 95%, hence *Z*_*a*_ = 1.96, and $Z_{a^{2}}$ = 3.84), p is the presumed prevalence of third-child fertility intentions (10%), (1 - p) is the proportion of intentions to not have a third child, and δ is the margin of error (δ = 0.02). Based on this formula, the minimum required sample size was 865. In addition, we factored in a nonresponse rate of 20%, which resulted in a sample size of 1,082.

### 2.4 Measurements

**Figure 1:**
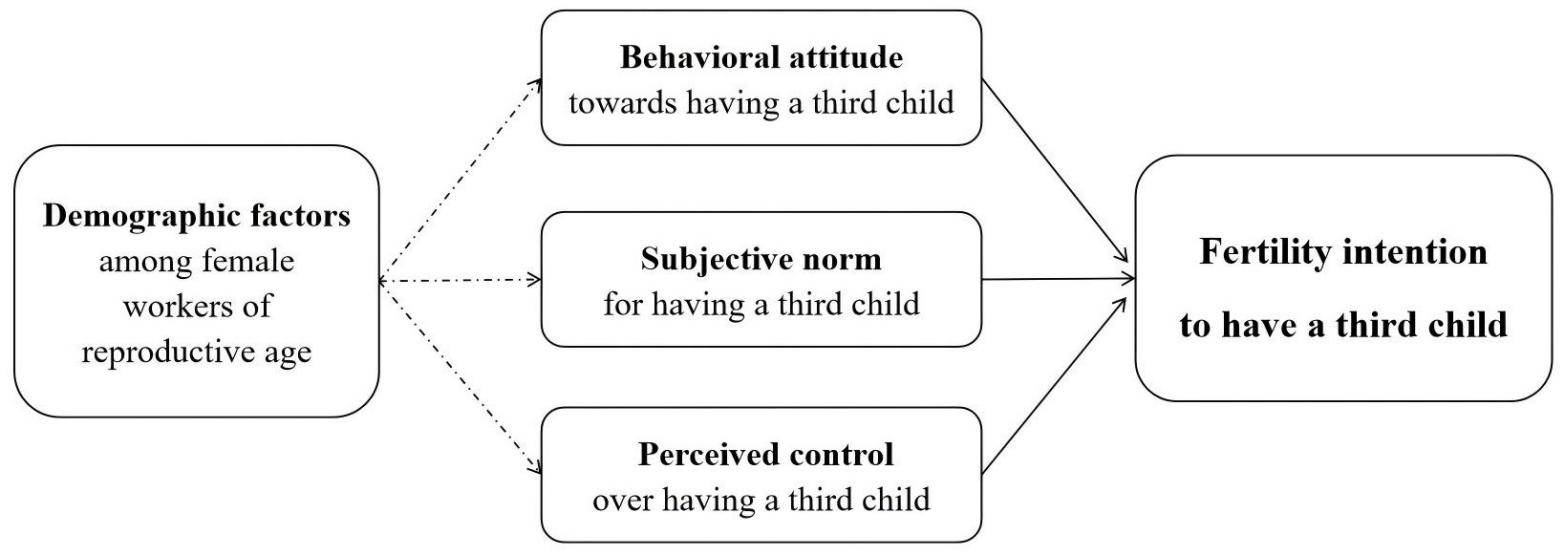
Theoretical framework.

We used the theory of planned behavior (TPB; Figure 1) to guide the identification of the factors that affected participants' fertility intentions. The theory emphasizes that behavioral intention is the most important factor determining whether or not to perform a behavior, and it is influenced by three factors: behavioral attitudes, subjective norms, and perceived control (18). Consequently, we assessed the following variables:

Demographic factors included *age* (0 = ≤ 29 years, 1 = 30–34 years, 2 = 35–39 years, 3 = ≥ 40 years), *ethnicity* (0 = Han, 1 = minority), *residence* (0 = city, 1 = rural), *education level* (0 = high school or below, 1 = specialized or bachelor's degree, 2 = master's degree or above), *monthly income* (0 = < 5,000 RMB, 1 = 5,000–10,000 RMB, 2 = > 10,000 RMB), outstanding *financial loans* (e.g., financial loans, housing loans, or car loans; 0 = yes, 1 = no), *whether both spouses were only children* (0 = both only children, 1 = only one an only child, 2 = both non-only children), *the number of current children* (0 = 0, 1 = 1, 2 = 2), and *having a son* (0 = yes, 1 = no; this question is not designed with gender-based value bias, but rooted in the realistic context of fertility decisions in Chinese society, aiming to objectively capture the potential impact of “gender structure preference” in traditional fertility culture).

We collected information on female workers' third-child fertility intentions by asking, “Do you intend to have a third child in practice?” The corresponding choices were “Yes” and “No.” If the study participants chose yes, they were asked to give the reasons for their third-child fertility intentions. To reduce the response burden on female workers, improve the efficiency of data collection and the quality of responses, while ensuring data standardization and facilitating statistical analysis through structured options, we adopted a multiple-choice format. The content of the multiple-choice questions included pressure from older adult(s) in the family, couples like to have more children, most friends and colleagues have three or more children, traditional concepts of fertility, current national welfare policy is good, more children mean children not being lonely when they grow up, and others (such as the poor health of the children they already had and the attitudes of their spouses).

The behavioral attitudes drawn from the TPB in this study referred to individuals' overall evaluations of childbearing, reflecting their positive or negative dispositions toward having more children. We captured them using the following four variables: *ideal number of children* (without considering various factors such as body, age, and economic status), *effect of delivery mode* (delivery mode refers to vaginal delivery and cesarean section two types; 0 = impact, 1 = no impact), *educational involution* (refers to a social phenomenon characterized by intense competition among families centered on their children's education, 0 = impact, 1 = no impact), and *career prospects* (0 = high impact, 1 = some impact, 2 = no impact).

The subjective norms drawn from the TPB in this study referred to individuals' perceptions of others' expectations and social pressures on their fertility behavior, including the influence of spouses, parents, and so on. We captured them using the following three variables: *couple's perception of fertility* (0 = consistent, 1 = inconsistent), *contribution of spouse* (include supportive behaviors in such aspects as sharing of childcare responsibilities, support in household chores, and emotional and decision-making support; 0 = impact, 1 = no impact), and *attitudes of parents* (0 = support, 1 = no idea, 2 = no support).

The perceived control factors drawn from the TPB in this study referred to individuals' perceptions of and confidence in their ability to support fertility behaviors, including resources and various types of social and financial support. We captured them using the following three variables: *caregiver for the third child* (0 = grandparents, 1 = childminder/childcare facility, 2 = the couple, 3 = others), *type of workplace* (0 = freelance, 1 = organization or institution, 2 = state-owned or collective enterprise, 3 = private or foreign-funded enterprise), and *knowledge of the three-child policy* (0 = very clear, 1 = know a little, 2 = heard about it, and 3 = never heard of it).

### 2.5 Data collection

We distributed questionnaires to teachers in the School of Nursing of Shandong Xiehe University; instructed the teachers to share the questionnaire link with family members, relatives, and so on; and adopted the snowball sampling method to further identify eligible survey respondents. We obtained informed consent from all the participants recruited for this study. We displayed an informed consent form on the first interface that participants saw when they opened the questionnaire link. If the participants clicked “Agree,” they could continue to answer the questions. If they clicked “Disagree,” they would be forced to exit the interface and could not proceed. Participants were also informed that they could withdraw from the study at any time and could contact the researchers if they had any questions about the questionnaire. The questionnaire was distributed using Wenjuanxing (www.wjx.cn)—a popular online survey platform in China—which allows surveys to be designed, distributed, and analyzed. The platform ensures questionnaire data quality through mandatory question settings and internet protocol address filtering. Following data collection, we implemented systematic data verification procedures. Questionnaires exhibiting certain response patterns, such as the uniform selection of identical options (e.g., all “A”) or repeated sequential responses (e.g., A → B → C → D) were systematically excluded to maintain data integrity.

### 2.6 Statistical analysis

We conducted all data analyses using SPSS 22.0 and described the respondents' general demographic characteristics and fertility intentions by frequency, rate (%), median, and interquartile range. *Third-child fertility intention* (yes/no) was taken as the dependent variable. We conducted univariate analysis of the factors influencing third-child fertility intentions using chi-squared tests. Factors demonstrating a statistically significant difference (*p* < 0.05) were subsequently included in a binary logistic regression model for multivariate analysis. To ensure the robustness of the logistic regression model and to rule out multicollinearity among the independent variables, we conducted multicollinearity diagnostics using the Variance Inflation Factor (VIF). In accordance with widely accepted criteria in statistical analysis (24), a VIF value of < 5 was considered indicative of no severe multicollinearity. We deemed statistical significance to be *p* < 0.05.

## 3 Results

### 3.1 Response rate and demographic characteristics of participants

Of the 1,394 returned questionnaires, we excluded 36 (2.58%) −23 (1.65%) due to incomplete responses and 13 (0.93%) due to repetitive selections (i.e., patterned responses such as uniform selection of identical options or sequential choices). After these exclusions, the final valid sample size was 1,358, and the effective response rate is 97.42%(1,358/1,394). The demographic characteristics of all participants are summarized in Table 1. The age range of the participants was 20–49 years, with a mean age of 30 years (range: 25–39). Most (80.3%) of the participants had specialized or bachelor's degrees or higher. Of the study participants, 40.8% reported a monthly household income exceeding 10,000 RMB ($1,407.2). The proportion of participants with zero, one, or two children was approximately balanced, with each group representing around 30% of the sample, with a larger proportion (61.6%) not having sons.

**Table 1:** Demographic characteristics of all participants (*N* = 1,358).

**Characteristics**	***n***	**%**
**Age (years)**		
≤ 29	622	45.8
30–34	201	14.8
35–39	213	15.7
≥40	322	23.7
**Ethnicity**		
Han	1,340	98.7
Minority	18	1.3
**Residence**		
City	1,082	79.7
Rural	276	20.3
**Education level**		
High school or below	267	19.7
Specialized or bachelor's degree	825	60.7
Master's degree or above	266	19.6
**Monthly household income (RMB)**		
< 5,000	214	15.8
5,000–10,000	590	43.4
>10,000	554	40.8
**Financial loan**		
Yes	682	50.2
No	676	49.8
**Whether both spouses are only children**		
Both only children	180	13.3
Only one an only child	745	54.8
Both non-only children	433	31.9
**Number of current children**		
0	525	38.7
1	395	29.1
2	438	32.2
**Having a son**		
Yes	522	38.4
No	836	61.6

### 3.2 Description of fertility intentions, behavioral attitudes, subjective norms, and perceived control factors

**Figure 2:**
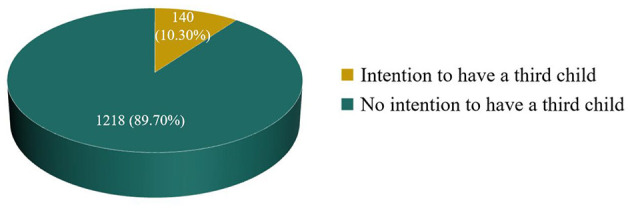
Third-child fertility intentions in practice among reproductive-age female workers (*N* = 1,358).

Of all the participants, 172 (12.7%) expressed a desire to have three or more children ideally, but only 140 (10.3%) intended to have a third child in practice (Figure 2).

**Figure 3:**
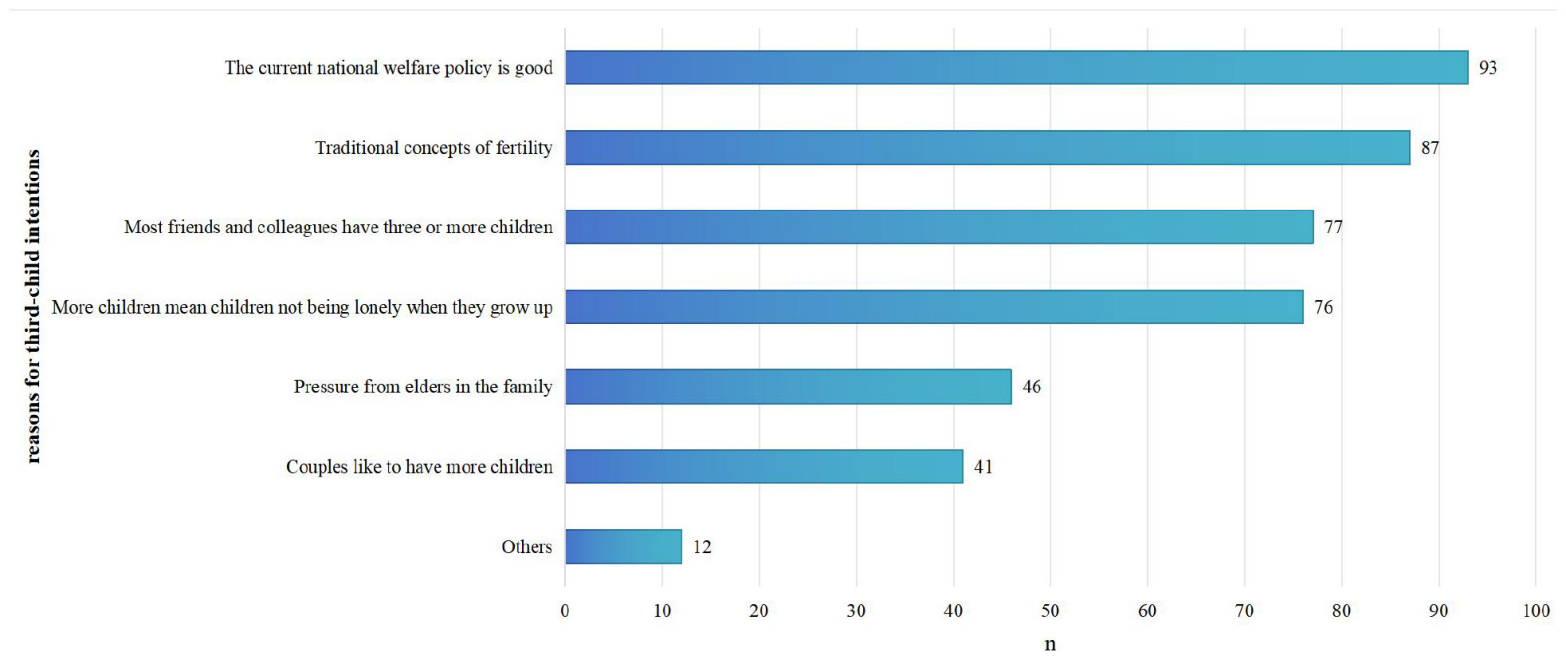
Reasons for intending to have a third child among the subset of female workers who expressed such intention (*n* = 140). Participants could select multiple reasons. Traditional fertility concepts included passing on the family name, having a son, raising children to support one's old age, and “the more children, the more happiness.” Others for third-child fertility intentions included the poor health of the children they already had and the attitudes of their spouses.

For the 140 respondents who indicated their intentions to have a third child (those who answered “Yes” to the question “Do you plan to have a third child?”), we used a multiple-choice question to investigate their reasons for desiring a third child, and 140 participants gave reasons for their third-child fertility intentions. Among these, the number of respondents who selected four of the reasons each accounted for more than 50% of the total number of respondents, the four reasons listed in descending order: current national welfare policy is good (93, 66.4%); traditional concepts of fertility, such as passing on the family name, having a son, raising children to support one's old age, and “the more children, the more happiness” (87, 62.1%); most friends and colleagues have three or more children (77, 55.0%), and more children mean children not being lonely when they grow up (76, 54.3%) (Figure 3).

The behavioral attitudes, subjective norms, and perceived control factors observed across all study participants are detailed in Table 2.

**Table 2:** Participants' behavioral attitudes, subjective norms, and perceived control factors (*N* = 1,358).

**Factors**	***n***	**%**
**Behavioral attitude factors**		
**Ideal number of children**		
0	78	5.7
1	365	26.9
2	743	54.7
≥3	172	12.7
**Mode of delivery**		
Impact	1,005	74.0
No impact	353	26.0
**Educational involution**		
Impact	1,136	83.7
No impact	222	16.3
**Career prospects**		
High impact	424	31.2
Some impact	805	59.3
No impact	129	9.5
**Subjective norm factors**		
**Couple's fertility perceptions**		
Consistent	1,188	87.5
Inconsistent	170	12.5
**Spouse's contribution**		
Impact	1,198	88.2
No impact	160	11.8
**Attitude of parents**		
Support	846	62.3
No idea	85	6.3
No support	427	31.4
**Perceived control factors**		
**Caregiver for the third child**		
Grandparents	759	55.9
Childminder/childcare facility	130	9.6
The couple	418	30.8
Others	51	3.7
**Type of workplace**		
Freelance	423	31.1
Organization or institution	387	28.5
State-owned or collective enterprise	213	15.7
Private or foreign-funded enterprise	335	24.7
**Knowledge of the three-child policy**		
Very clear	233	17.2
Know a little	471	34.7
Heard about it	534	39.3
Never heard of it	120	8.8

Behavioral attitude factors: these factors reflect individuals' overall evaluations of childbearing, indicating positive or negative dispositions toward having a third child, as per the Theory of Planned Behavior (TPB). Ideal number of children: the number of children a participant would like to have under ideal circumstances, without considering practical constraints such as health, age, economic status, or policy limitations. Effect of delivery mode: whether the mode of delivery (vaginal delivery or cesarean section) for previous births influences the decision to have a third child. Educational involution: refers to the perceived impact of “educational involution” (a social phenomenon characterized by intense competition among families centered on children's education, leading to high parental investment and stress) on fertility intentions. Career prospects: the perceived influence of having a third child on the participant's career development and professional advancement. Subjective Norm Factors: these factors represent individuals' perceptions of social pressures and expectations from significant others (e.g., spouse, parents) regarding fertility behavior, as per TPB. Couple's perception of fertility: whether the couple shares consistent views on fertility and childbearing decisions. Contribution of spouse: the perceived level of spouse's involvement and support in childcare responsibilities, household chores, and emotional or decision-making aspects. Attitudes of parents: the level of support or pressure from parents (or in-laws) regarding the decision to have a third child. Perceived Control Factors: these factors reflect individuals' perceptions of their ability to control and manage the resources and challenges associated with having a third child, as per TPB. Caregiver for the third child: the anticipated primary caregiver for the third child, indicating resource availability and support systems. Type of workplace: the type of employment sector or workplace environment, which influences work-life balance, job stability, and access to family-friendly policies. Knowledge of the three-child policy: the level of awareness and understanding of China's three-child policy, including its incentives, subsidies, and support measures.

In terms of *behavioral attitude factors*, 743 (54.7%) reported a preferred family size of two children. Of the total participants, 90.5% (1,229/1,358) felt that career prospects would affect their desire to have a third child.

In terms of *subjective norm factors*, 87.5% (1,188/1,358) of the participants reported that the fertility perceptions of the couple were consistent, 88.2% (1,198/1,358) indicated that spousal contributions had an impact on their third-child fertility intentions, and 62.3% (846/1,358) had parents who supported them in having a third child.

In terms of *perceived control factors*, 30.8% (418/1,358) of the participants reported that childcare responsibility for their third child would fall solely on the couple, 31.1%(423/1,358) were freelancers. The proportion of participants who were very well-informed about the three-child policy was relatively small (17.2%, 233/1,358).

### 3.3 Factors affecting fertility intentions

The results demonstrated statistically significant differences in fertility intentions across the variables aligned with the Theory of Planned Behavior (TPB) framework (Figure 1).

*Demographic characteristics: age* (*x*^2^ = 49.31, *p* < 0.001), *ethnicity* (*x*^2^ = 8.09, *p* = 0.004), *residence* (*x*^2^ = 7.74, *p* = 0.005), *education level* (*x*^2^ = 17.42, *p* < 0.001), *monthly household income* (*x*^2^ = 49.87, *p* < 0.001), *whether both spouses are only children* (*x*^2^ = 48.01, *p* < 0.001), and *having a son* (*x*^2^ = 8.42, *p* = 0.004).

*Behavioral attitude factors*: *ideal number of children* (*x*^2^ = 163.75, *p* < 0.001) and *career prospects* (*x*^2^ = 39.98, *p* < 0.001).

*Subjective norm factors*: *couple's fertility perceptions, spouse's contribution*, and *parents' attitudes* (all TPB-based subjective norm variables) showed no statistically significant association with third-child intentions (*p* > 0.05).

*Perceived control factors*: *caregiver for the third child* (*x*^2^ = 62.13, *p* < 0.001), *type of workplace* (*x*^2^ = 34.53, *p* < 0.001), and *knowledge of the three-child policy* (*x*^2^= 65.24, *p* < 0.001).

The full statistical details are presented in Table 3.

**Table 3:** Univariate analysis of third-child fertility intentions among female workers (*N* = 1,358).

**Characteristics**	**Yes (*N* = 140) *n* (%)**	**No (*N* = 1,218) *n* (%)**	***x* ^2^**	***p*-Value**
**Demographic characteristics**				
**Age (years)**				
≤ 29	100 (16.1)	522 (83.9)	49.31	< 0.001^*^
30–34	21 (10.4)	180 (89.6)		
35–39	10 (4.7)	203 (95.3)		
≥40	9 (2.8)	313 (97.2)		
**Ethnicity**				
Han	134 (10.0)	1,206 (90.0)	8.09	0.004^*^
Minority	6 (33.3)	12 (66.7)		
**Residence**				
City	99 (9.1)	983 (90.9)	7.74	0.005^*^
Rural	41 (14.9)	235 (85.1)		
**Education level**				
High school or below	38 (14.2)	229 (85.8)	17.42	< 0.001^*^
Specialized or bachelor's degree	92 (11.2)	733 (88.8)		
Master or above	10 (3.8)	256 (96.2)		
**Monthly household income (RMB)**				
< 5,000	12 (5.6)	202 (94.4)	49.87	< 0.001^*^
5,000–10,000	32 (5.4)	558 (94.6)		
>10,000	96 (17.3)	458 (82.7)		
**Financial loan**				
Yes	72 (10.6)	610 (89.4)	0.09	0.763
No	68 (10.1)	608 (89.9)		
**Whether both spouses are only children**				
Both only children	38 (21.1)	142 (78.9)	48.01	< 0.001^*^
Only one an only child	41 (5.5)	704 (94.5)		
Both non-only children	61 (14.1)	372 (85.9)		
**Number of current children**				
0	58 (11.0)	467 (89.0)	1.01	0.603
1	42 (10.6)	353 (89.4)		
2	40 (9.1)	398 (90.9)		
**Having a son**				
Yes	38 (7.3)	484 (92.7)	8.42	0.004^*^
No	102 (12.2)	734 (87.8)		
**Behavioral attitude factors**				
**Ideal number of children**				
0	2 (2.6)	76 (97.4)	163.75	< 0.001^*^
1	18 (4.9)	347 (95.1)		
2	55 (7.4)	688 (92.6)		
≥3	65 (37.8)	107 (62.2)		
**Mode of delivery**				
Impact	96 (9.6)	909 (90.4)	2.40	0.122
No impact	44 (12.5)	309 (87.5)		
**Educational involution**				
Impact	121 (10.7)	1,015 (89.3)	0.88	0.348
No impact	19 (8.6)	203 (91.4)		
**Career prospects**				
High impact	16 (3.8)	408 (96.2)	39.98	< 0.001^*^
Some impact	96 (11.9)	709 (88.1)		
No impact	28 (21.7)	101 (78.3)		
**Subjective norm factors**				
**Fertility perceptions of the couple**				
Consistent	117 (5.9)	1,071 (53.7)	2.18	0.140
Inconsistent	23 (13.5)	147 (86.5)		
**Spouse's contribution**				
Impact	123 (10.3)	1,075 (89.7)	0.02	0.889
No impact	17 (10.6)	143 (89.4)		
**Attitude of parents**				
Support	89 (10.5)	757 (89.5)	0.47	0.792
No idea	10 (11.8)	75 (88.2)		
No support	41 (9.6)	386 (90.4)		
**Perceived control factors**				
**Caregiver for the third child**				
Grandparents	96 (12.6)	663 (87.4)	62.13	< 0.001^*^
Childminder/childcare facility	31 (23.8)	99 (76.2)		
The couple	8 (1.9)	410 (98.1)		
Others	5 (9.8)	46 (90.2)		
**Type of workplace**				
Freelance	45 (10.6)	378 (89.4)	34.53	< 0.001^*^
Organization or institution	54 (14.0)	333 (86.0)		
State-owned or collective enterprise	33 (15.5)	180 (84.5)		
Private or foreign-funded enterprise	8 (2.4)	327 (97.6)		
**Knowledge of the three-child policy**				
Very clear	50 (21.5)	183 (78.5)	65.24	< 0.001^*^
Know a little	64 (13.6)	407 (86.4)		
Heard about it	22 (4.1)	512 (95.9)		
Never heard of it	4 (3.3)	116 (96.7)		

^*^p-Value < 0.05.

### 3.4 Results of the logistic regression analysis

Table 4 presents the analysis of independent variables. The multicollinearity diagnostics revealed that the Variance Inflation Factor (VIF) values for all independent variables ranged from 1.018 to 1.286 (all below 2), confirming the absence of multicollinearity issues in the regression model. Table 5 displays the binary logistic regression results-with variables categorized according to the TPB framework (Figure 1) to clarify how each TPB dimension influences third-child intentions.

**Table 4:** Assignment table.

**Variables**	**Assignment**
**Demographic characteristics**	
Age	≤ 29 = 0, 30–34 = 1, 35–39 = 2, ≥40 = 3
Ethnicity	Han = 0, minority = 1
Residence	City = 0, rural = 1
Education level	High school or below = 0, specialized or bachelor's degree = 1, master's degree or above = 2
Monthly household income (RMB)	< 5,000 = 0, 5,000–10,000 = 1, >10,000 = 2
Whether both spouses are only children	Both only children = (0, 0, 0), Only one an only child = (0, 1, 0), both non-only children = (0, 0, 1)
Having a son	Yes = 0, no = 1
**Behavioral attitude factors**	
Ideal number of children	0 = 0, 1 = 1, 2 = 2, ≥3 = 3
Career prospects	High impact = 0, some impact = 1, no impact = 2
**Perceived control factors**	
Caregiver for the third child	Grandparents = ( 0, 0, 0, 0), childminder/childcare facility = (0, 1, 0, 0), the couple = (0, 0, 1, 0), others = (0, 0, 0, 1)
Type of workplace	Freelance = (0, 0, 0, 0), organization or institution = (0, 1, 0, 0), state-owned or collective enterprise = (0, 0, 1, 0), private or foreign-funded enterprise = (0, 0, 0, 1)
Knowledge of the three-child policy	Very clear = 0, know a little = 1, heard about it = 2, never heard of it = 3

**Table 5:** Logistic regression analysis for factors affecting third-child fertility intentions.

**Variables**	**Beta coefficient**		***Waid* *x*^2^**	***p*-Value**	**OR**	**95% CI**
	***B***	**st. error**				
Constant	−4.527	1.424	10.100	0.001	0.011	/
**Demographic characteristics**						
Age	−0.863	0.140	38.163	0.000	0.422	[0.321, 0.555]
Residence	0.755	0.307	6.068	0.014	2.128	[1.167, 3.880]
Education level	−1.765	0.289	37.423	0.000	0.171	[0.097, 0.301]
Monthly household income	1.246	0.208	35.785	0.000	3.475	[2.311, 5.226]
**Whether both spouses are only children**						
Both only children	Reference					
Only one an only child	−1.206	0.364	10.981	0.001	0.300	[0.147, 0.611]
Both non-only children	−0.094	0.359	0.068	0.794	0.911	[0.450, 1.841]
**Behavioral attitudes factors**						
The ideal number of children	1.211	0.176	47.201	0.000	3.356	[2.376, 4.470]
Career prospects	0.881	0.217	16.462	0.000	2.414	[1.577, 3.696]
**Perceived control factors**						
**Caregiver of the third child**						
Grandparents	Reference					
Childminder/childcare facility	−0.002	0.334	0.000	0.996	0.998	[0.519, 1.922]
The couple	−2.129	0.457	21.730	0.000	0.119	[0.049, 0.291]
Others	−1.212	0.763	2.522	0.112	0.298	[0.067, 1.328]
**Type of workplace**						
Freelance	Reference					
Organization or institution	0.108	0.333	0.106	0.745	1.114	[0.580, 2.140]
State-owned or collective enterprise	0.343	0.369	0.861	0.353	1.409	[0.683, 2.905]
Private or foreign-funded enterprise	−1.194	0.498	5.739	0.017	0.303	[0.114, 0.805]
Knowledge of the three-child policy	−0.932	0.154	36.660	0.000	0.394	[0.291, 0.533]

OR, odds ratio; CI, confidence interval.

*In terms of demographic characteristics*, third-child fertility intentions decreased significantly as age and education level increased (*OR* = 0.422, *95% CI* = 0.321–0.555; *OR* = 0.171, *95% CI* = 0.097–0.301). In contrast, third-child fertility intentions increased significantly with a higher monthly household income and an increase in the number of desired children (*OR* = 3.475, *95% CI* = 2.311–5.226; *OR* = 3.356, *95% CI* = 2.376–4.470). People living in rural areas (*OR* = 2.128, *95% CI* = 1.167–3.880) were more likely to have a third child than those living in urban areas. Couples with only one spouse who was an only child (*OR* = 0.300, *95% CI* = 0.147–0.611) were less likely to have a third child than couples with both spouses who were only children.

In terms of *behavioral attitude factors*, the greater the ideal number of children and the lower the level of influence of career prospects, the higher the working women's third-child fertility intentions (*OR* = 2.414, *95% CI* = 1.577–3.696).

In terms of *perceived control factors*, the likelihood of having a third child was lower for couples who would have to care for the third child themselves (*OR* = 0.119, *95% CI* = 0.049–0.291) than for those whose third child would be cared for by its grandparents. Compared to freelancers, women working in private and foreign companies (*OR* = 0.303, *95% CI* = 0.114–0.805) had lower third-child fertility intentions. The lower the participants' knowledge of the three-child policy, the lower their third-child fertility intentions (*OR* = 0.394, *95% CI* = 0.291–0.533).

## 4 Discussion

### 4.1 Main study findings

Under the three-child policy, three-child fertility intentions among women of reproductive age have become a key societal issue. A survey conducted among 1,358 female workers of reproductive age revealed that only 140 individuals (10.3%) actually intended to have a third child. This finding underscores a substantial challenge for current population policies. The four most significant reasons cited were that the current national welfare policy was good (66.4%), traditional concepts of fertility (62.1%), most friends and colleagues had three or more children (55.0%), and more children meant that the children would not be lonely when they grew up (54.3%). A logistic regression analysis of the factors influencing these women's third-child fertility intentions revealed that *demographic characteristics*, such as age, residence, education level, monthly household income, and the couple's family situation, significantly affected their three-child fertility intentions. The ideal number of children and career prospects, as *behavioral attitude factors*, were also influential. Additionally, *perceived control factors*, such as the caregiver for the third child, the type of workplace, and knowledge of the three-child policy, also affected their fertility intentions. Interestingly, *subjective norm factors* did not emerge as influencing factors in the final models.

### 4.2 Status of third-child fertility intentions among female workers of reproductive age

Third-child fertility intentions among our sample (10.3%) are consistent with the 8%−12.2% range reported in studies of East Asian female workers (25–27), but lower than the 20.2% in general reproductive-age women (28). This gap underscores the unique burden of work-family balance for female workers. Compared to Hainan's 8% intention rate (25), Shandong's slightly higher rate may be attributed to two context-specific factors: first, Shandong's Confucian “more children, more blessings” culture (cited by 62.1% of our participants) is stronger, and second, our sample reported higher awareness of national policy support (cited by 66.4% of our participants), reflecting more effective policy promotion in Shandong. In contrast to studies by Yan et al. (27) (12.2%) and Jing et al. (23) (15.1%), our lower intention rate stems from our exclusive focus on female workers: unlike general reproductive-age women, they face career interruption risks and workplace discrimination post-childbirth (14, 17), which dampen fertility willingness—a mechanism supported by Pan et al.'s (14) qualitative study on work-family conflict in Chinese female workers.

### 4.3 Factors influencing third-child intentions among female workers of reproductive age

Our findings demonstrate that *demographic characteristics*, such as age, residence, education level, monthly household income, and couple's family situation, significantly predict fertility intentions among female workers. Existing studies have confirmed that increasing age, living in cities, and lower monthly household income significantly deter women from having a third child (11, 20, 23, 29).

Highly educated female workers showed lower third-child intentions. The TPB posits that attitudes toward childbearing are influenced by perceived costs and benefits. Highly educated women may perceive the opportunity costs of childbearing as prohibitive, given their investment in career advancement. This is consistent with the perspectives of previous studies showing that compared to participants with a high school education, the third-child fertility intentions of those with bachelor's and master's degrees decreased by 37.7 and 44.6%, respectively (27). This finding is noteworthy because it highlights the role of gender equality and career aspirations in shaping fertility decisions (30). However, unlike previous studies, our research focused on female workers, with highly educated female workers tending to prioritize career development and financial independence, aligning with the broader trend of modernization and shifting gender roles in China. Furthermore, their reliance on pension systems rather than on children for old-age support reduced the perceived necessity of having additional children (31). Another study has confirmed that for individuals with higher education levels, the number of siblings exerts a stronger negative impact on trust in the government, thereby indirectly reducing their fertility intentions (32). Consistent with the findings of this study, this jointly verifies that education level is an important predictor of people's fertility intentions and strongly validates the applicability of the Theory of Planned Behavior (TPB) in explaining this demographic trend. These insights suggest that policies promoting gender equality and career development complementarity could be effective in addressing low fertility intentions among highly educated female workers.

Our findings indicate that third-child fertility intentions among female workers are significantly influenced by family structure. Families with only one spouse who is an only child are less likely to have a third child than dual only-child couples, a pattern consistent with observations from Central China under similar policy contexts (20).This discrepancy can be attributed to the distribution of caregiving responsibilities and resource allocation within extended families. Dual only-child couples benefit from consolidated support networks that alleviate childcare-related economic burdens and opportunity costs. In contrast, families with only one only-child spouse face fragmented intergenerational support, leading to insufficient assistance for both childcare and care for older adults (33). This highlights the importance of family-supportive policies, such as community-based childcare services and targeted interventions for dual only-child couples to enhance their capacity for work–family reconciliation and fertility intention optimization.

Of the demographic characteristics examined, the factor of “Having a son” warrants particular attention. In contrast to the findings reported by Jiang et al. (7), our univariate analysis indicated that women without a son demonstrated a significantly higher intention to have a third child. However, this association became non-significant in the binary logistic regression model. This discrepancy suggests that the influence of son preference among female workers may be indirect or conditional. First, the initial association observed in the univariate analysis might reflect confounding effects from resource-related variables. Second, female workers may prioritize practical feasibility over traditional gender norms. Supporting this, studies have shown that among urban employed women, such preferences are moderated by the availability of structural support and resources. This observation aligns with the broader conclusion that modernization and increased female labor force participation are attenuating traditional gender-based motivations for fertility (34).

Based on the TPB, a positive behavioral attitude serves as a driving force that facilitates a behavior, whereas a negative attitude exerts an inhibitory effect on behavioral execution. Our study showed that, in terms of *behavioral attitudes*, the ideal number of children and career prospects were factors influencing third-child fertility intentions among female workers. The less significant the impact of childbearing on women's career development, the more inclined they were to have a third child, it is consistent with the findings of related studies (31). This phenomenon indicates that female workers hold negative attitudes toward having a third child, as childbearing may lead to restricted career development, including fewer opportunities for job promotion and an increased risk of career interruption.

An interesting finding is that the important factors influencing female workers' third-child fertility intentions in present-day China are related to the ideal number of children, while the number of current children has no effect. The positive correlation between the ideal number of children and fertility intentions highlights that female workers have positive attitudes toward having a third child, which typically stems from their perception of the benefits of multi-child families, such as a livelier family atmosphere, stronger emotional bonds, mutual support among children, and fulfilling the traditional cultural expectation of “more children, more blessings” (19, 35). This result strongly underscores the positive influence of the ideal number of children aligns with *TPB's behavioral attitude*, reflecting a positive evaluation of a large family. But in reality, their third-child fertility intentions is low. The reason for this obvious discrepancy may be that although female workers identify with traditional family values, they cannot break free from the constraints of the workplace and practical resources on fertility decisions, resulting in a fertility mindset of “wanting to but daring not to.” About the number of current children did not affect the participants' fertility intentions. The essence of this differential phenomenon is a disconnect between value-driven motivations and realistic constraints, possibly related to childbearing costs, maternal penalties, and the emphasis on childrearing quality over quantity. We recommend that future researchers further explore this discrepancy using mixed methods to better understand how female workers balance ideal family visions with practical constraints so that fertility intentions can truly translate into childbearing behavior.

An unexpected result was that a couple's fertility perceptions, the spouse's contribution, and the attitude of parents were the three *subjective norm factors* that had no impact on female workers' third-child fertility intentions. This finding deviates from the predictions of the Theory of Planned Behavior (TPB) framework and contradicts the results reported by Ning et al. (4) and Chen et al. (20). The possible reasons for this may be attributed to several interconnected factors. Firstly, measurement issues may have played a role, as the constructs of subjective norms, such as spouse's contribution and parents' attitudes were assessed using single-item measures. This approach might have failed to capture nuanced behaviors, such as specific spousal involvement in nocturnal child care or educational activities, or the intensity of parental support (e.g., verbal encouragement vs. tangible help in childcare), potentially leading to an underestimation of their true effects. Future studies could adopt multi-item scales to enhance measurement precision. Secondly, socio-cultural and structural factors offer another explanation. The lack of significant findings may reflect broader trends in China, such as the rise of nuclear families, which accounted for 55.3% of households according to the 2020 National Census (36). In this context, fertility decisions among highly educated female workers (80.3% holding associate degrees or higher) may rely more on personal autonomy and spousal negotiation than on traditional parental pressure (37). Additionally, structural constraints like financial limitations, competitive workplace environments, and insufficient policy support may diminish the role of subjective norms, making practical resources the important factors in shaping fertility intentions (28). For instance, the absence of a significant association between spousal contribution and female workers' intention to have a third child may be attributed to their greater reliance on systemic support mechanisms, the constraints of entrenched gender roles, and their own high decision-making thresholds. Finally, model specification issues may also be relevant. It is possible that the influence of subjective norms operates indirectly through perceived behavioral control, a mediating pathway not examined in this study. Further analysis might reveal that subjective norms enhance perceived control, which in turn affects fertility decisions, suggesting the need for future research to explore such mediated relationships. Furthermore, the (TPB) model can be further adjusted to adapt to the practical context where individual autonomy surpasses traditional social pressures in highly private decisions such as childbearing.

In the Theory of Planned Behavior (TPB), *perceived control*—an individual's subjective assessment of behavioral feasibility—bridges attitude and action, critical for explaining fertility intentions (3, 11). Our analysis identifies three perceived control dimensions shaping female workers' third-child intentions: childcare responsibility, workplace type, and three-child policy familiarity, aligning with and extending prior TPB research. Consistent with Ning et al. (4), who highlighted childcare support as core to perceived control, we find reliance on spousal-only childcare (vs. grandparental support) reduces fertility willingness. This underscores that childcare control is a psychological barrier: sole couple responsibility amplifies burden, especially for employed women balancing work and family (14, 23). Our finding that workplace type (private/foreign enterprises vs. freelancing) affects perceived control echoes Pan et al. (17), who linked rigid workplace norms to reduced work-childbearing balance. Unlike freelancers with schedule autonomy (20), private enterprise workers report lower time control, suppressing intentions (28). Aligning with Li et al. (30), policy familiarity boosts perceived feasibility. For workers, it provides resources and reduces uncertainty, critical amid workplace pressures where unclear policies heighten career penalty risks (16). This study revealed that only 17.2% of the respondents had a “very clear” understanding of the three-child policy, while 82.8% demonstrated insufficient or complete lack of knowledge. This finding highlights a discernible gap between policy promotion and its actual comprehension among the target population. These consistencies (4, 17, 20, 30) reinforce TPB's utility, while our focus on employed women reveals unique mechanisms: their perceived control hinges on family-work interplay, not just attitudes. Interventions should strengthen grandparental support, workplace flexibility, and policy outreach to boost third-child intentions.

### 4.4 Analysis of the significance implications of the study

The issue of declining global fertility rates is a multifaceted social phenomenon that encompasses various factors, including economic, cultural, and social policies. In this study, we examined the current state and key influencing factors of third-child fertility intentions among female workers of reproductive age under China's three-child policy. Fertility intentions for a third child were found to be notably low within the studied cohort, a trend which may exacerbate the risk of falling into a “low fertility trap,” where persistently low birth rates lead to a shrinking future labor force, thereby increasing pressures on pension systems and sustained economic growth. Furthermore, these findings suggest that existing policy measures may be insufficient to address the underlying causes of low fertility within this specific demographic. Therefore, interventions informed by the influencing factors identified in this study are crucial. While the immediate context is Shandong Province, China, the implications are broader. We argue that, on a global scale, particularly in countries and regions sharing socio-economic and cultural backgrounds similar to Shandong Province, China (such as other Chinese provinces, South Korea, certain parts of Japan, and northern Vietnam), effective measures can be implemented to reduce economic burdens, balance work–family pressures, and strengthen social support systems, thereby encouraging more families to have children. Addressing the decline in fertility rates necessitates a holistic policy approach, as well as a gradual shift in cultural attitudes, to effectively increase fertility rates worldwide and mitigate the challenges posed by population aging.

### 4.5 Limitations

Despite its contributions, this study has several limitations that should be acknowledged, along with concrete suggestions for future research to address these gaps: *First*, the snowball sampling method, while practical for accessing a hard-to-reach population, may introduce selection bias, potentially leading to an over-representation of views from specific professional networks (e.g., healthcare, academia). Furthermore, the exclusive focus on Shandong Province, despite its significant cultural relevance as the birthplace of Confucianism, limits the direct generalizability of the findings to all female workers across China's diverse socioeconomic regions. Future research should therefore employ stratified random sampling techniques to ensure a more nationally representative cohort of female workers. Specifically, large-scale, multi-region comparative studies are needed—contrasting findings from economically developed (e.g., Guangdong, Zhejiang), developing (e.g., Henan, Anhui), and western (e.g., Sichuan, Gansu) provinces—to truly disentangle the influence of regional economic and cultural factors on fertility intentions. *Second*, as a cross-sectional study, this research provides a valuable baseline snapshot of associations but cannot establish causality or elucidate dynamic processes and mediating mechanisms underlying fertility decision-making. To address this, future studies should implement longitudinal panel designs, tracking the evolution of fertility intentions among the same cohort of female workers over time (e.g., across 3–5 years) in response to life events, career progression, and policy changes. Furthermore, interventional or quasi-experimental studies, perhaps evaluating the impact of newly introduced corporate or local government family-support policies (e.g., subsidized childcare, extended parental leave) on fertility intentions, could provide stronger evidence for causal relationships. *Third*, the reliance on single-item measures for complex constructs (e.g., “career prospects,” “spouse's contribution”) represents a significant limitation, as these measures lack the depth and reliability of validated multi-item scales and may fail to capture nuanced behaviors and attitudes. Combined with the use of self-reported data, this potentially introduces measurement error and biases related to social desirability. Future research must prioritize the use of validated, psychometrically robust instruments. For instance, constructs like ‘spousal contribution' could be measured using adapted scales assessing tangible support (e.g., sharing of nocturnal childcare, participation in educational activities) and emotional support. Employing mixed-methods designs—integrating quantitative surveys with in-depth qualitative interviews—would be particularly valuable for deepening our understanding of the subjective experiences and decision-making processes of female workers, thereby enhancing the overall validity and richness of findings. *Fourth*, while the use of a dichotomous (Yes/No) response format simplifies the analysis of fertility intentions, it may compel participants to provide definitive answers when they are genuinely uncertain. Future research should adopt more nuanced Likert-scale measures to capture the degree of certainty (e.g., from “very unlikely” to “very likely”) and explicitly include an “undecided” or “uncertain” option. Furthermore, a conditional branching logic question, such as “If you are uncertain, which factors most influence your decision? (e.g., “unclear policy benefits,” “unstable childcare support”),” could be implemented for those selecting the “undecided” option. Subsequently, latent class analysis (LCA) can be employed to identify distinct subgroups within the “uncertain” population. Finally, logistic regression analysis can track how these subgroups transition toward “intending” or “not intending” over time, revealing the dynamic pathways of fertility decision-making.

## 5 Conclusion

In this study, we examined the current status and influencing factors of fertility intentions among female workers of reproductive age under the three-child policy in Shandong Province, China. We found that three-child fertility intentions are currently low within this population. Key factors influencing third-child fertility intentions included age, residence, education level, monthly household income, couple's family situation, number of ideal children, caregiver for the third child, career prospects, type of workplace, and knowledge of the three-child policy. The findings indicate that governmental efforts should focus on enhancing the promotion of the three-child policy, extending financial and childcare support, and legislating flexible work arrangements to foster family-friendly environments. These measures are crucial for promoting population growth in Shandong Province and optimizing China's demographic structure. This study offers important implications for population development in regions and countries sharing socio-economic and cultural backgrounds similar to Shandong Province, China.

## Data Availability

The original contributions presented in the study are included in the article/supplementary material, further inquiries can be directed to the corresponding author.
